# *Clocks & Sleep*: A New Open-Access Journal to Publish Your Circadian and Sleep Research Results

**DOI:** 10.3390/clockssleep1010001

**Published:** 2018-04-18

**Authors:** Christian Cajochen, Paul Franken

**Affiliations:** 1 Psychiatric Hospital of the University of Basel, Centre for Chronobiology, Wilhelm-Kleinstr. 27, CH-4002 Basel, Switzerland; 2 University of Lausanne, Center for Integrative Genomics, Genopode Building, CH-1015 Lausanne-Dorigny, Switzerland

## Why *Clocks & Sleep*?

Exciting new discoveries in the circadian and sleep field have mushroomed in the past 10 years, culminating in the 2017 Nobel Prize in Physiology or Medicine being awarded to Jeffrey C. Hall, Michael Rosbash, and Michael W. Young for their discoveries of molecular mechanisms controlling the circadian rhythm. According to PubMed, 43% of the total 245,281 publications containing the words “circadian or sleep”, were published between March 2008 and March 2018. These figures impressively demonstrate how fast and active a young research field is branching out in all aspects of life ranging from circadian genes and molecules, to sleepy cells, circadian rhythms, and sleep in organs and different organisms and superorganisms, as well as in the field of circadian and sleep disorders, in epidemiology, in developing wearables and nearables, and in the computational modeling of circadian regulation and sleep-wake states. With this new open-access journal, *Clocks & Sleep*, we aim to integrate all of these fascinating facets. Thus, we structured the journal in many different sections with corresponding section editors in an attempt to anticipate the wishes of potential submitting authors.

## Why Would You Submit Your Work to *Clocks & Sleep*?

Despite a sizable number of already existing scientific journals in the field, we can think of several reasons why you should consider publishing in *Clocks & Sleep*:

1. We are convinced that true progress in our field can be made only by integrating sleep AND circadian aspects. While circadian topics can be found in our leading sleep journals and publications concerning sleep can be found in leading circadian journals, they each remain largely underrepresented in the respective categories (*SLEEP* and the *Journal of Sleep Research* have “circadian” in 2–3% of titles and in 17–19% of all search items; the *Journal of Biological Rhythms* exhibits “sleep” in 7% of titles and in 19% of all search items, according to PubMed), showing that both fields are still considered separate. *Clocks & Sleep* strives for a more balanced and integrative approach and is particularly interested in contributions bridging this gap.
2. We have been successful in putting together an amazing editorial board comprising more than 50 members who reflect a fair mix between very established and senior researchers, as well as more junior representatives covering very different aspects in the *Clocks & Sleep* field. In fact, we were very surprised about how fast and motivated people were to respond to our invitation to join the board. Their support enhances our confidence in the project and shows that this new platform is welcomed by the community. In order to keep track of current trends in sleep and circadian research, the composition of the editorial board will be updated on a regular basis.
3. The journal offers a fair, fast, rigorous, and transparent peer review method through a collaborative process between the submitting authors, editors, and professional staff at MDPI.
4. Besides open-access (free access) and being free of charge to publish in the first two years, *Clocks & Sleep* also provides different publication formats including short communications, regular reports, reviews, commentaries, and the publication of pre-registered studies or trials.
5. Besides publishing fascinating findings, reviews, and commentaries in the circadian and sleep area, *Clocks & Sleep* will also support young research scientists with travel grants, awards for the best publications, as well as support meetings and educational programs in the field.
6. *Clocks & Sleep* is open to all aspects of circadian and sleep research and welcomes submissions to the following sections:
  - C & S in animal basic research
  - C & S in human basic research
  - C & S and computational models
  - C & S and methodology and technology
  - C & S and zeitgeber
  - C & S and development
  - C & S and society
  - C & S and disorders
  - C & S and preregistered trials
7. *Clocks & Sleep* accepts so-called Registered Reports. This new publication format aims to refocus on high quality science instead of ‘good’ results in an effort to change research practice towards improved reproducibility and reliability of biomedical science.

We hope you will find the new open-access journal *Clocks & Sleep* to be a new and fresh alterative to publish your results in a fast but still rigorous and transparent peer review process, demanding high standards. Together, we can make *Clocks & Sleep* a dynamic and high quality open access circadian/sleep journal with the greatest possible reach and research impact!

